# Dexamethasone as additive of local infiltration analgesia reduces opioids consumption after simultaneous bilateral total hip or knee arthroplasty: a randomized controlled double-blind trial

**DOI:** 10.1186/s13018-023-04164-y

**Published:** 2023-09-22

**Authors:** Dasai Wang, Wang Chen, Leshu Zhang, Zhigang Wang, Yu Qian, Tao Li, Jianning Sun

**Affiliations:** 1 Orthopedic Center, Nanjing Jiangbei Hospital, Nanjing, 210043 Jiangsu People’s Republic of China; 2 Department of Orthopedics Surgery, The First Affiliated Hospital of Wannan Medical College, Wuhu, 241000 Anhui People’s Republic of China; 3 Department of Orthopedics Surgery, The Second Affiliated Hospital of Harbin Medical University, Harbin, 150000 Heilongjiang People’s Republic of China; 4 Department of Orthopedics Surgery, Nanjing Drum Tower Hospital Group Suqian Hospital, Suqian, 223800 Jiangsu People’s Republic of China; 5 Department of Orthopedics Surgery, The Affiliated Hospital of Xuzhou Medical University, Xuzhou, 221000 Jiangsu People’s Republic of China

**Keywords:** Hip, Knee, Dexamethasone, Analgesia, Opioids

## Abstract

**Purpose:**

A randomized controlled double-blind trial was conducted to evaluate the effects of adding dexamethasone to the local infiltration analgesia (LIA) mixture on frequency of patient controlled analgesia (PCA) and opioids consumption after simultaneous bilateral total hip or knee arthroplasty (THA or TKA).

**Methods:**

108 patients who received simultaneous bilateral THA or TKA were randomly divided into dexamethasone group and normal saline (NS) group. The main difference between two groups was whether or not dexamethasone was added to the LIA mixture. The main outcome was the cumulative consumption of opioids within 24 h. The secondary outcome were the total cumulative consumption of opioids during postoperative hospitalization, consumption of opioids drug for rescue analgesia, frequency of PCA, postoperative Visual Analogue Scale (VAS), and complications.

**Results:**

Cumulative consumption of opioids in the 24 h was similar between two groups (*P* = 0.17). Total cumulative consumption of opioids in the dexamethasone group during postoperative hospitalization was significantly lower (*P* = 0.03). No significant difference in the consumption of opioids drug for rescue analgesia between two groups within 24 h, while the frequency of PCA was significantly different (*P* = 0.04). VAS of dexamethasone group and NS group were similar during postoperative hospitalization, while the incidence of postoperative nausea and vomiting (PONV) in dexamethasone group was lower than that in NS group.

**Conclusions:**

Adding dexamethasone to LIA in the simultaneous bilateral THA or TKA can effectively reduce the total cumulative consumption of opioids and the frequency of PCA, as well as reduce the incidence of PONV.

*Trial Registration* The trial has been registered in the Chinese Clinical Trial Registry (Registration Number: ChiCTR2100042551, Date: 23/01/2021).

## Introduction

For patients with advanced stage bilateral hip or knee arthritis, simultaneous bilateral total joint arthroplasty (TJA) can obtain higher partial benefits than staged bilateral TJA, especially in terms of time and cost [1, 2]. At the same time, it brings more challenges to orthopedic surgeons, due to the difficulty and risk of analgesia of simultaneous bilateral TJA [3, 4]. Simultaneous bilateral total hip or knee arthroplasty (THA or TKA) has a higher degree of postoperative pain, and an immediate and effective analgesia has been shown to help improve postoperative clinical outcomes, promote rapid recovery, and reduce opioid-related side-effects [5, 6]. Therefore, the selection of reasonable analgesic methods for surgery is particularly important to improve the prognosis of patients.

Multimodal analgesia has been widely used in recent years, encompassing patient controlled analgesia (PCA), epidural analgesia, femoral nerve block (FNB), and local infiltration analgesia (LIA) [7, 8]. PCA can effectively control postoperative pain, however, it may also cause subsequent side-effects such as postoperative nausea and vomiting (PONV), constipation, and respiratory depression [9]. Epidural analgesia increases the risk of nausea, hypotension, and respiratory depression [10]. Although FNB has been reported to have a good analgesic effect, it can increase quadriceps weakness and increase the risk of falls in the hospital, especially in patients after joint arthroplasty [11]. In contrast, LIA has the advantages of convenient use, good analgesic effect, and few side effects [12]. Studies have shown that LIA was also beneficial to functional recovery after THA or TKA and to promote early getting out of bed [13]. However, the limited duration of local analgesia is a shortcoming of LIA [14]. On the basis of the advantages of LIA, improving its duration of action will be more conducive to postoperative analgesia and improve patient satisfaction. However, it is still unclear whether the addition of adjuvants to the LIA mixture can prolong the effective duration of local anesthesia in bilateral THA or TKA.

Glucocorticoids have powerful anti-inflammatory effects and are widely used in various perioperative environments to reduce inflammation markers and hospital stay, prevent PONV, and relieve postoperative pain and fatigue [15, 16]. However, it is difficult to judge the actual value of glucocorticoids due to the heterogeneity of the type, dosage, dosing schedule, and perioperative treatment during joint arthroplasty [16, 17]. Previous studies have proved that dexamethasone as an additive of LIA mixture can play a role in short-term analgesia and moderate improvement of postoperative function, hospital stay, and nausea during unilateral THA or TKA [18, 19]. Although simultaneous bilateral TJA has many advantages, it is also accompanied by heavier postoperative pain and the use of opioids [3, 20]. The effect of dexamethasone as a LIA mixture additive in simultaneous bilateral THA or TKA has not been studied yet. We hypothesized that dexamethasone as a LIA mixture additive would have better advantages in simultaneous bilateral THA or TKA. A randomized controlled double-blind study was conducted to evaluate the effects of adding dexamethasone to the LIA mixture on opioid consumption, frequency of PCA, short-term pain scores, and complications after selective primary simultaneous bilateral THA or TKA.

## Materials and methods

### Trial design

A prospective, double-blind, parallel-group, randomized controlled clinical trial was conducted. The trial has been registered in the Chinese Clinical Trial Registry (Website: https://www.chictr.org.cn/, Registration Number: ChiCTR2100042551, Register Date: 23/01/2021). This study was approved by the Ethics Committee of the Hospital (Ethics Number: AF/SC-08/02.0). The work has been reported in line with Consolidated Standards of Reporting Trials (CONSORT) Guidelines. Before grouping, each patient signed an informed consent form and agreed to participate.

### Participants

Inclusion criteria:

1. The American Society of Anesthesiologists (ASA) classification: I–III
2. BMI range of 18–40 kg/m^2^
3. People aged 18–85 who can communicate normally and effectively without cognitive behavioral impairments
4. Selective primary simultaneous bilateral THA or TKA that operated by a participant surgeon were enrolled

Exclusion criteria:

1. Allergy or intolerance to one of the study drugs
2. Regular use of opioids, or addiction to opioids and alcohol
3. Suffering from pain-causing diseases such as neuritis and herpes
4. Severe spine and ankle joint diseases that affect the function of the knee or hip
5. Prior hip or knee surgery, previous hip or knee infection
6. Insufficiency of liver or kidney
7. Rheumatoid arthritis, ankylosing spondylitis
8. Long-term use of glucocorticoids or recent intra-articular steroid injection

### Perioperative management

All patients were given 60 mg Etocoxib tablets daily for analgesia 3 days before surgery. Surgical anesthesia used general anesthesia without regional block and were completed by anesthesiologists proficient in general anesthesia. General anesthesia were induced in the induction room using anesthesia machine face mask inhalation induction method (Sufentanil Citrate Injection, 50 ug/ml, 0.1–5.0 ug/kg for opioids). Gradually increase the inhalation concentration during the induction of anesthesia, and when the patient's consciousness disappears and enter the third phase of anesthesia, intratracheal intubation can be performed by intratracheal infusion of muscle relaxants. The maintenance of general anesthesia (Remifentanil Hydrochloride for Injection, 50 ug/ml, 0.5–1 ug/kg for opioids) was maintained by continuous injection of intravenous anesthetics in the operating room to meet the requirements of the operation and the management of the patient, and to ensure the stability of physiological functions such as circulation and breathing.

Operations were performed by the same team of experienced surgeons using the same surgical method. Tranexamic Acid and Sodium Chloride Injections (0.5 g, 3 vessel) were injected intravenously before the skin incision to reduce perioperative blood loss. After waiting for the implantation of the intraoperative prosthesis, the surgeon implemented LIA (200 mg 1% Ropivacaine Hydrochloride Injection, 100 mg Flurbiprofen Axetil Injection, 1 mg Epinephrine, 0.9% normal saline (NS)) and injected the mixture into muscles and subcutaneous tissues to ensure complete coverage of the joint structure [21]. Superficial and deep injections were performed in the late stage of surgery (just after implantation) [22]. The main difference between the two groups was that 1 ml of 0.9% NS was added to the LIA mixture for each joint in the NS group, while the dexamethasone group was 1 ml of 5 mg/ml dexamethasone. Place the drainage tube and suture the incision. After the operation, the drainage tube was clamped for 2 h and opened for 10 min. No long-acting opioids were used during the operation. The patient did not receive intravenous dexamethasone at other times during the perioperative period.

### Postoperative management

After the TJA, the patient were transferred to the postanesthesia care unit (PACU) for postoperative vital signs monitoring. Propofol (4–12 mg/kg) was used for postoperative anesthesia maintenance. Metoclopramide Hydrochloride Injection (10 mg/ml, 10–20 mg) was used for nausea and vomiting, Naloxone Hydrochloride Injection (1 mg/ml, 0.4–2 mg) for respiratory depression, and Diphenhydramine Hydrochloride Tablets (25–50 mg, tid) for itchy skin. The breathing tube was removed when the patient recovered to the Aldrete score of 9 points, and the patients were returned to the ward after 15 min of observation for no abnormalities [23].

Two groups of patients received the same dose of Flurbiprofen Axetil Injection (50 mg, twice a day) intravenously and NSAID Etoricoxib tablets (120 mg/day) orally for intensive analgesia. All patients used patient controlled intravenous analgesia (PCIA) containing opioid drug Sufentanil Citrate Injection (2 ug/kg, 100 ml) and 0.9% NS with a self-control switch device as the PCA plan. The quantitative sustained release background dose of PCIA was 2 ml/h. When the analgesia was insufficient [19], press the automatic switch device. The switch can only be pressed once within 15 min, and the pulse dose was 0.5 ml per pulse. Opioid drug Dezocine (5 mg/ml) was used for postoperative rescue analgesia.

The postoperative rehabilitation programs were the same for both groups. The rehabilitation physician instructed the patients to perform quadriceps and hamstring exercises preoperatively and on the second postoperative day.

## Outcome measures

### Primary outcome measure

The main outcome measure was the cumulative consumption of opioids measured by morphine dose equivalent (MDE) within 24 h after arriving at the PACU. All opioids were converted to MDE according to the standard conversion table [24, 25].

### Secondary outcome measures

The total cumulative consumption of opioids was measured in terms of MDE during hospitalization. And, the MDE of opioid drug Dezocine, which was used for rescue analgesia within 24 h after surgery.

Record the time point of each PCA self-controlled medication for all patients. The automatic control switch of PCA can only be pressed once in 15 min. Calculate the frequency of PCA in every 2 h interval (maximum 8 self-controlled pressings) within 24 h after operation.

Visual Analogue Scale (VAS) at rest and during activity at preoperation, PACU, postoperative day (POD) 0, 1, 2, and 3. VAS usually uses a 10-cm long straight line, with 0 points and 10 points at the two ends, 0 means no pain, 10 means the most unbearable pain, and the middle value means different degrees of pain. The patient makes a mark on a certain point on the straight line to indicate the intensity of the pain and the psychological impact [26].

Record opioid-related side-effects such as vomiting and itching, and surgical complications, including wound complications, thromboembolic diseases, infections, etc., within 24 h.

### Sample size

Cumulative MDE consumption within 24 h was the main result of the study. Based on experience and institutional pre-experimental data, the mean ± standard deviation (SD) dose of MDE in patients who underwent simultaneous bilateral TJA and did not use dexamethasone within 24 h after surgery was 78.6 (58.3) mg. In order to prove that the addition of dexamethasone to LIA can reduce the consumption of opioids within 24 h by 30% [27], 48 patients in each group need to detect the statistically significant difference between the alpha value of 0.05% and the power of 80%. Considering about 10% of incomplete follow-ups or withdrawal of patients, our goal was to recruit a total sample size of 108 patients.

### Randomization and blinding

After obtaining the patient’s informed consent, the statistician used computer to generate a 1 to 108 randomization sequence (www.randomization.com), and assigned the patients in a 1-to-1 ratio to one of the following two groups in parallel: dexamethasone group (1 mL of dexamethasone 5 mg/mL), and NS group (1 mL of NS 0.9%). The patients must be explained about scope of trial and outcome measurement before enrolling. All outcome measures were performed by professionally trained evaluators, who do not know the grouping and treatment plan of the patients throughout the study. In addition, patients, anesthesiologists, physical therapists, and nurses do not know the content of the trial group and intervention measures.

### Statistical analysis

SPSS 26.0 (Chicago, IL, USA) statistical software was used to process the data, and general data were statistically described using frequency and percentage. Chi-squared test was used for binary variables. After the continuous variables were tested by Shapiro-Wilk test, the data with normal distribution were used independent-sample t-test between the two groups, expressed as the mean ± SD. Data conversion was performed on the data with non-normal distribution, and the data with normal distribution after the conversion adopted the independent sample t test, and the data without the normal distribution after the conversion adopted the Mann–Whitney U test, which was expressed as median (IQR (range)). Multiple measurements at different time points were compared using ANOVA of repeated measurements. All tests were calculated with two tails, *P* < 0.05 indicated a statistically significant difference between the two groups.

## Result

### Patient characteristics and flow

**Fig. 1:**
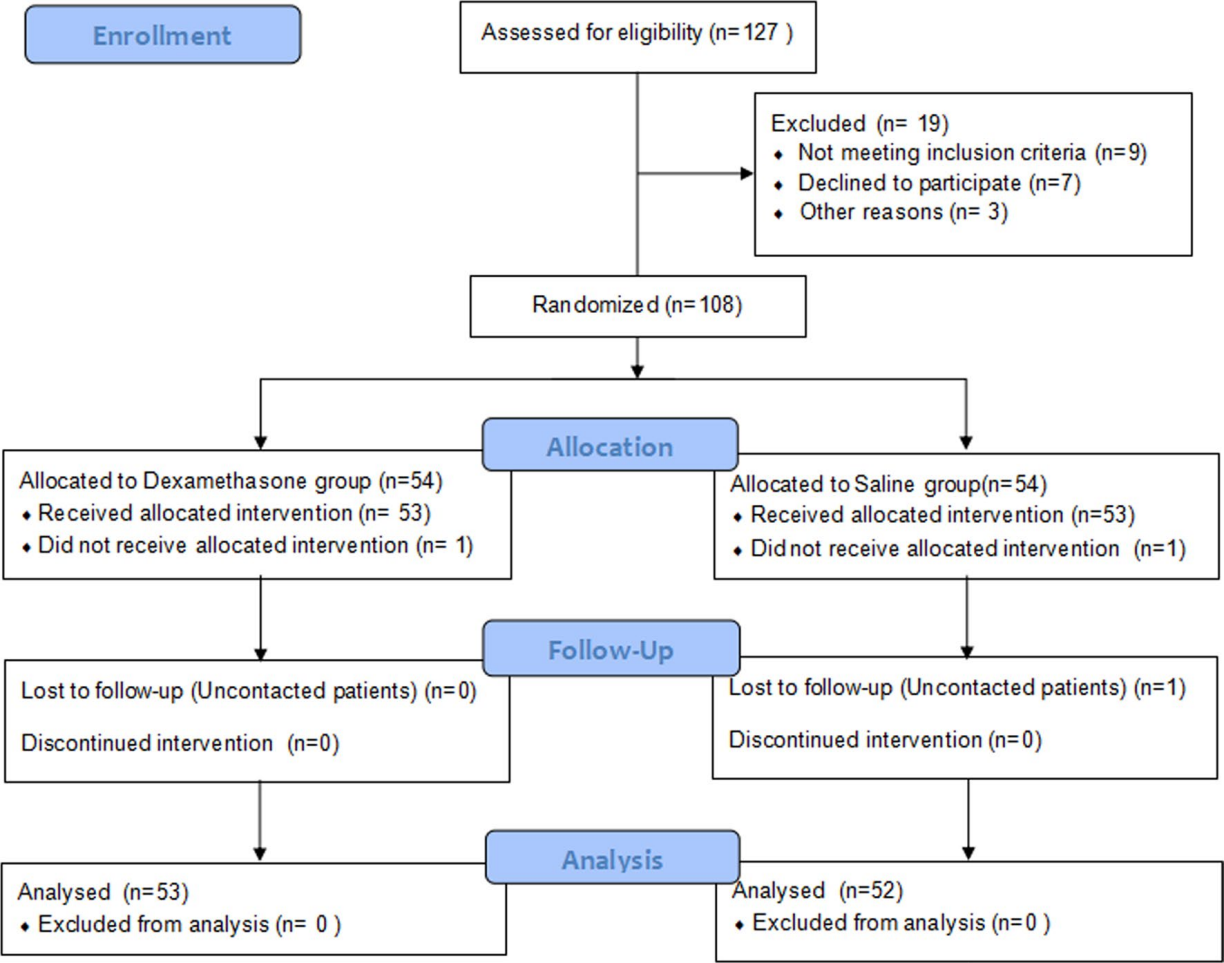
The detailed enrollment, randomization flow diagram of the patient

We performed simultaneous bilateral THA or TKA on 127 patients. According to the inclusion and exclusion criteria, 108 patients were screened out and randomized (one to one ratio). 1 patient was transferred to other departments for treatment due to acute illness and dropped out, and 2 patients were transferred to the intensive care unit after surgery. Finally, the complete data of 105 patients were obtained, 53 patients in the dexamethasone group and 52 patients in the NS group. The detailed flow chart of patient enrollment and randomization is shown in Fig. 1.

Statistical analysis proved that there was no statistical difference in the composition of baseline demographic data such as gender, age, BMI, and surgical category between the two groups. For a detailed comparison of the demographic characteristics of the two groups of patients, see Table 1.

**Table 1:** Preoperative baseline demographic data of patients between dexamethasone and NS group

	Dexamethasone (*n* = 53)	Range	95% CI	NS (*n* = 52)	Range	95% CI	*P* value
Gender (male/female)	21/32			22/30			0.15^†^
Age (years)	61.5 ± 12.5	29.0–87.0	(58.1, 65.0)	63.1 ± 12.4	31.0–81.0	(59.7, 66.6)	0.52^*^
Weight (kg)	62.9 ± 6.9	47.8–78.7	(61.0, 64.7)	63.5 ± 7.3	36.0–79.0	(61.5, 65.6)	0.62^*^
BMI (kg/m^2^)	23.4 ± 3.1	15.8–30.3	(22.6, 24.3)	24.0 ± 3.4	16.5–31.5	(23.0, 24.9)	0.44^*^
Types of surgery (%)							0.14^†^
TKA	32 (60.4)			34 (65.4)			
THA	21 (39.6)			18 (34.6)			
History of diabetes (%)	12 (22.6)			13 (25.0)			0.17^†^
VAS at rest	2.7 ± 0.8	1.1–4.2	(2.4, 2.9)	2.8 ± 1.0	0.8–5.0	(2.5, 3.1)	0.59^*^
VAS during activity	5.3 ± 1.4	2.6–7.8	(4.9, 5.6)	5.6 ± 1.5	2.9–8.1	(5.2, 6.0)	0.20^*^
Duration of surgery (min)	170.3 ± 18.9	126.0–203.0	(165.1, 175.5)	171.4 ± 18.1	141.0–212.0	(166.4, 176.5)	0.75^*^

*NS* normal saline; *CI* confidence interval; *BMI* body mass index; *TKA* total knee arthroplasty; *THA* total hip arthroplasty; *VAS* visual analogue scale

^*^P value means that student’s *t* test were used, data were represented as mean ± SD

^†^*P* value indicates that the Chi-squared test were used

### Outcome measures

In the main outcome measure, the consumption of MDE in the NS group and dexamethasone group within 24 h was similar (mean ± SD (95% confidence interval (CI)), 74.3 ± 21.8 (68.2, 80.3) and 68.8 ± 19.4 (63.4, 74.1) mg, respectively, *P* = 0.17), and without statistical difference **(**Table 2**)**.

**Table 2:** Morphine dose equivalent (MDE) (mg) between dexamethasone and NS group

Time	Dexamethasone (*n* = 53)	Range	95% CI	NS (*n* = 52)	Range	95% CI	*P* Value^*^
Within 24 h	68.8 ± 19.4	23.7–103.1	(63.4, 74.1)	74.3 ± 21.8	33.3–113.2	(68.2, 80.3)	0.17
Within 48 h	111.7 ± 35.9	36.7–185.7	(101.8, 121.6)	124.9 ± 35.4	41.6–177.0	(115.0, 134.7)	0.06
Within 72 h	119.6 ± 36.6	41.9–195.2	(109.5, 129.7)	133.7 ± 39.8	46.2–211.9	(122.6, 144.8)	0.06
Total	130.2 ± 38.6	47.2–198.5	(119.6, 140.9)	147.6 ± 42.1	53.8–232.7	(135.9, 159.3)	0.03^**^
Dezocine							
Within 24 h	5.2 ± 2.2	0–10	(4.6, 5.8)	5.7 ± 2.2	0–10	(5.1, 6.3)	0.26

*NS* normal saline; *CI* confidence interval

*P value means that student’s t test were used, data were represented as mean ± SD

**The difference was statistically significant

In the secondary outcome measures, the total cumulative consumption of opioids during the postoperative hospitalization was significantly lower in dexamethasone group than in NS group (mean ± SD (95% CI), 130.2 ± 38.6 (119.6, 140.9) and 147.6 ± 42.1 (135.9, 159.3) mg, respectively, *P* = 0.03), and the MDE of the opioid drug Dezocine used for rescue analgesia within 24 h after the operation of the two groups were not significantly different (*P* = 0.26) **(**Table 2**)**.

**Fig. 2:**
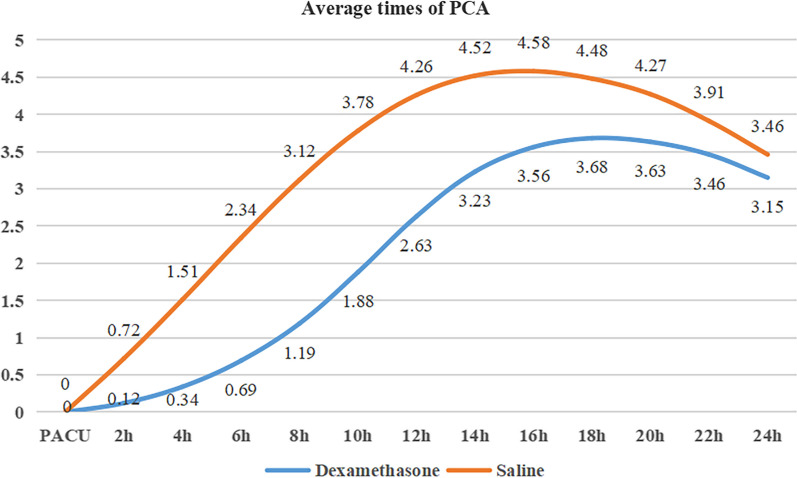
Average times of PCA between dexamethasone and NS group

Figure 2 shows the frequency of PCA every 2 h in the two groups within 24 h after surgery. The total number of times in the dexamethasone and the NS group were 27.56 and 40.95 times (*P* = 0.04), with a significant difference between the two groups.

**Fig. 3:**
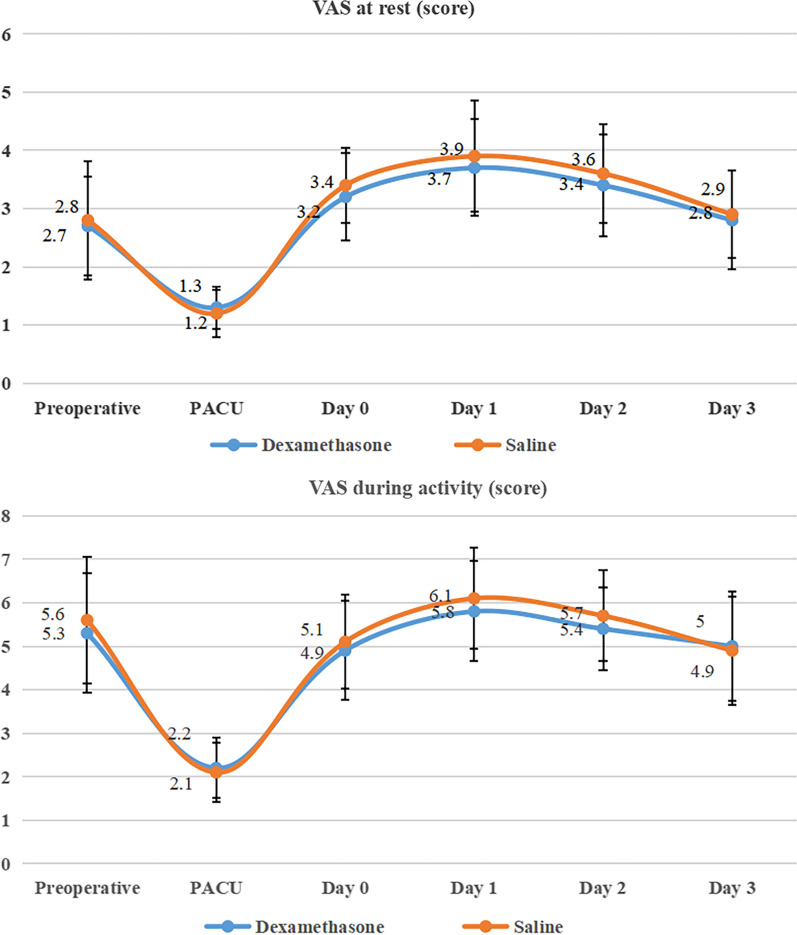
VAS at rest and during activity between dexamethasone and the NS group

The short-term VAS of pre-operation, PACU, and POD 0–3 were shown in Fig. 3. The graphs of the two groups of VAS at rest (Fig. 3a) and during activity (Fig. 3b) had similar trends and without significant difference at each time point.

The incidence of PONV in patients treated with dexamethasone decreased (*P* < 0.05). No significant difference in opioid-related side-effects and surgical complications **(**Table 3**)**.

**Table 3:** Opioid-related side-effects and surgical complications between dexamethasone and NS group

	Dexamethasone (*n* = 53)	NS (*n* = 52)	*P* value^†^
Opioid-related complications			
Nausea			
Day 0	12 (22.6)	23 (44.2)	0.01^**^
Day 1	11 (20.8)	19 (36.5)	0.04^**^
Vomiting			
Day 0	8 (15.1)	15 (28.8)	0.05^**^
Day 1	9 (17.0)	10 (19.2)	0.19
Vertigo			
Day 0	3 (5.7)	4 (7.7)	0.28
Day 1	1 (1.9)	1 (1.9)	0.5
Urinary retention			
Day 0	3 (5.7)	2 (3.8)	0.32
Day 1	1 (1.9)	1 (1.9)	0.5
Pruritus			
Day 0	5 (9.4)	6 (11.5)	0.23
Day 1	4 (7.7)	7 (13.5)	0.16
Over-sedation or somnolence			
Day 0	1 (1.9)	2 (3.8)	0.37
Surgical complications			
Wound complications	1 (1.9)	2 (3.8)	0.37
Thromboembolic disease	1 (1.9)	0 (0)	0.5
Superficial infection	2 (3.8)	1 (1.9)	0.38

*NS* normal saline

Data are represented as n (%)

^†^*P* value indicates that the Chi-squared test were used

**The difference between the two groups was statistically significant

## Discussion

This trial was the first prospective double-blind randomized controlled clinical trial to test the analgesic effect of dexamethasone on LIA in simultaneous bilateral THA or TKA. The results showed that although the addition of dexamethasone to LIA does not reduce the consumption of opioids within 24 h after simultaneous bilateral THA or TKA and the consumption of opioid drug Dezocine for rescue analgesia after surgery, it can effectively reduce the total consumption of opioids during hospitalization and the frequency of PCA within 24 h. The results of the study also concluded that there was no significant difference in VAS at rest and during activity between the dexamethasone group and the NS group at each time point, however, it could effectively alleviate the side effects of opioids such as PONV.

This study was a prospective double-blind randomized controlled clinical trial, and also the first trial to study simultaneous bilateral THA or TKA, which will be different from unilateral THA or TKA. Several clinical trials have reported the benefit of adding dexamethasone to the LIA mixture in patients undergoing TJA [16–19]. Summarizing the previous literature and our study, the benefit of dexamethasone in TJA is significant, therefore, dexamethasone as additive of LIA is recommended for orthopedic surgery. Our data provide a valuable addition to the literature and may go some way toward deepening our understanding of the effects of dexamethasone.

The sources of pain included nerve stimulation and inflammatory response [28]. After joint arthroplasty, multiple inflammatory factors including IL-1β, IL-6, transforming growth factor-α (TGF-α) were significantly increased [29]. The release of a large amount of inflammatory mediators can lower the nociceptor threshold and cause pain sensitization [30], so that patients will experience severe pain after receiving mild stimulation. Simultaneous bilateral TJA has greater trauma and releases more inflammatory mediators, and patients often suffer from higher pain and analgesic doses after surgery [31], including the application of opioids. The results of the study proved that the addition of dexamethasone to LIA in simultaneous bilateral THA or TKA can effectively reduce the total consumption of opioids during postoperative hospitalization, which was similar to the results reported in previous studies in the addition of dexamethasone to LIA during unilateral THA or TKA [32, 33]. The reduction in the total consumption of opioids can increase the safety of arthroplasty and reduce the risks associated with anesthetics [34, 35]. El-Boghdadly et al. conducted a randomized controlled trial of adding dexamethasone to LIA during selective total hip arthroplasty, and they believed that adding dexamethasone to LIA in unilateral total hip arthroplasty can effectively reduce VAS on the POD 1, and that this conclusion was related to the time of action of dexamethasone [18]. We have concluded that there was no significant difference in VAS at rest and during activity at each time point between the NS group and the dexamethasone group, which may be related to the use of opioid drug Sufentanil Citrate Injection analgesia pump with automatic switching device as PCA regimen in the study, and related to the addition of opioid drug Dezocine for postoperative rescue analgesia when the analgesia was insufficient. The decrease in the incidence of PONV in the dexamethasone group suggested that LIA may have a systemic mechanism of dexamethasone [19]. The application of dexamethasone in this study did not increase surgical complications during hospitalization and could even effectively reduce opioid-related side-effects, which indirectly proves the effectiveness and safety of dexamethasone.

We used the PCIA with a quantitative continuous release background dose and a compression-controlled pulsed dose, and recorded the frequency of PCA every 2 h in the two groups within 24 h after surgery in this study. The consumption of opioids in the dexamethasone group and the NS group (66.8 vs. 74.3 mg) within 24 h was the sum of the quantitative sustained release background dose (48.0 vs. 48.0 mg), rescue analgesic Dezocine and compression controlled pulse dose. The study concluded that the use of rescue analgesia opioid drug Dezocine within 24 h was not significantly different between the two groups (5.2 vs. 5.7 mg, *P* = 0.26). Therefore, the difference in the cumulative consumption of opioids between the two groups within 24 h was mainly due to the pressure-controlled pulse dose. The study showed that the total average times of the dexamethasone group and the NS group was 27.56 and 40.95 times, and the patients in the NS group had a higher frequency of PCA within 24 h, and with significant difference. Higher frequency of self-control may mean higher negative emotions such as psychological anxiety. Negative emotions, such as anxiety and depression, can exacerbate pain that is difficult to overcome with painkillers [36]. The interaction of pain and negative emotions will increase the psychological and physical burden of patients, affect the postoperative recovery of patients, and reduce surgical satisfaction [37]. As far as we know, this was the first study to include the frequency of PCA self-controlled dosing and obtain a positive result, which was not noticed in previous studies.

This study also has several shortcomings: First, we do not know the optimal dose of dexamethasone added to LIA, and the addition of dexamethasone 5 mg to LIA in this study was determined based on previous studies [31, 38], which was a safe dose [39]. Second, for the convenience of trial calculation, we converted all opioids in the study to MDE, which may be inaccurate. Third, the LIA mixture included Ropivacaine Hydrochloride Injection, Flurbiprofen Axetil Injection, Epinephrine, etc., which means that we cannot rule out the effects of other adjuvants and dexamethasone compatibility. Fourth, patients’ preoperative NSAID use cannot be controlled and predicted, which may have affected the results of this study.

In conclusion, adding dexamethasone to LIA can reduce the total cumulative consumption of opioids and the frequency of PCA during the postoperative hospital stay, as well as reduce the incidence of PONV symptoms in patients, however, we did not find that dexamethasone can reduce 24 h opioid consumption and relieve short-term pain scores. More accurate studies on simultaneous bilateral THA or TKA need to be carried out to verify the accuracy of the results.

## Data Availability

The datasets used and/or analyzed during the current study available from the corresponding author on reasonable request.

## References

[1] Odum SM, Troyer JL, Kelly MP, Dedini RD, Bozic KJ (2013). A cost-utility analysis comparing the cost-effectiveness of simultaneous and staged bilateral total knee arthroplasty. J Bone Jt Surg Am.

[2] Partridge TCJ, Charity JAF, Sandiford NA, Baker PN, Reed MR, Jameson SS (2020). Simultaneous or staged bilateral total hip arthroplasty? An analysis of complications in 14,460 patients using national data. J Arthroplasty.

[3] Tsukada S, Wakui M, Hoshino A (2015). Pain control after simultaneous bilateral total knee arthroplasty: a randomized controlled trial comparing periarticular injection and epidural analgesia. J Bone Jt Surg Am.

[4] Morcos MW, Hart A, Antoniou J, Huk OL, Zukor DJ, Bergeron SG (2018). No difference in major complication and readmission rates following simultaneous bilateral vs unilateral total hip arthroplasty. J Arthroplasty.

[5] Burton BN, Padwal JA, Swisher MW, Salinas CR, Gabriel RA (2019). Postoperative outcomes with neuraxial versus general anesthesia in bilateral total hip arthroplasty. J Clin Anesth.

[6] Laoruengthana A, Rattanaprichavej P, Rasamimongkol S, Galassi M (2017). Anterior vs posterior periarticular multimodal drug injections: a randomized, controlled trial in simultaneous bilateral total knee arthroplasty. J Arthroplasty.

[7] Yadeau JT, Goytizolo EA, Padgett DE, Liu SS, Mayman DJ, Ranawat AS, Rade MC, Westrich GH (2013). Analgesia after total knee replacement: local infiltration versus epidural combined with a femoral nerve blockade: a prospective, randomised pragmatic trial. Bone Jt J.

[8] Albrecht E, Guyen O, Jacot-Guillarmod A, Kirkham KR (2016). The analgesic efficacy of local infiltration analgesia vs femoral nerve block after total knee arthroplasty: a systematic review and meta-analysis. Br J Anaesth.

[9] Grass JA (2005). Patient-controlled analgesia. Anesth Analg.

[10] Sitsen E, van Poorten F, van Alphen W, Rose L, Dahan A, Stienstra R (2007). Postoperative epidural analgesia after total knee arthroplasty with sufentanil 1 microg/ml combined with ropivacaine 0.2%, ropivacaine 0.125%, or levobupivacaine 0.125%: a randomized, double-blind comparison. Reg Anesth Pain Med.

[11] Paul JE, Arya A, Hurlburt L, Cheng J, Thabane L, Tidy A, Murthy Y (2010). Femoral nerve block improves analgesia outcomes after total knee arthroplasty: a meta-analysis of randomized controlled trials. Anesthesiology.

[12] Seangleulur A, Vanasbodeekul P, Prapaitrakool S, Worathongchai S, Anothaisintawee T, McEvoy M, Vendittoli P-A, Attia J, Thakkinstian A (2016). The efficacy of local infiltration analgesia in the early postoperative period after total knee arthroplasty: a systematic review and meta-analysis. Eur J Anaesthesiol.

[13] Zhao J, Davis SP (2019). An integrative review of multimodal pain management on patient recovery after total hip and knee arthroplasty. Int J Nurs Stud.

[14] Kehlet H, Andersen LØ (2011). Local infiltration analgesia in joint replacement: the evidence and recommendations for clinical practice. Acta Anaesthesiol Scand.

[15] Tolska HK, Hamunen K, Takala A, Kontinen VK (2019). Systematic review of analgesics and dexamethasone for post-tonsillectomy pain in adults. Br J Anaesth.

[16] Xu B, Ma J, Huang Q, Huang Z-Y, Zhang S-Y, Pei F-X (2018). Two doses of low-dose perioperative dexamethasone improve the clinical outcome after total knee arthroplasty: a randomized controlled study. Knee Surg Sports Traumatol Arthrosc.

[17] Lei Y-T, Xu B, Xie X-W, Xie J-W, Huang Q, Pei F-X (2018). The efficacy and safety of two low-dose peri-operative dexamethasone on pain and recovery following total hip arthroplasty: a randomized controlled trial. Int Orthop.

[18] El-Boghdadly K, Short AJ, Gandhi R, Chan V (2021). Addition of dexamethasone to local infiltration analgesia in elective total knee arthroplasty: double-blind, randomized control trial. Reg Anesth Pain Med.

[19] El-Boghdadly K, Short AJ, Gandhi R, Chan VWS (2019). Addition of dexamethasone to local infiltration analgesia in elective total hip arthroplasty: a double-blind, randomized control trial. Reg Anesth Pain Med.

[20] Triantafyllopoulos GK, Fiasconaro M, Wilson LA, Liu J, Poeran J, Memtsoudis SG, Poultsides LA (2020). Bilateral total knee arthroplasty and in-hospital opioid dispension: a population-based study. J Arthroplasty.

[21] Sun J-N, Chen W, Zhang Y, Zhang Y, Feng S, Chen X-Y (2020). Does cognitive behavioral education reduce pain and improve joint function in patients after total knee arthroplasty?. A Randomized Controll Trial Int Orthop.

[22] Busch CA, Shore BJ, Bhandari R, Ganapathy S, MacDonald SJ, Bourne RB, Rorabeck CH, McCalden RW (2006). Efficacy of periarticular multimodal drug injection in total knee arthroplasty. A randomized trial. J Bone Jt Surg Am.

[23] Steinthorsdottir KJ, Awada HN, Abildstrøm H, Kroman N, Kehlet H, Aasvang EK (2020). Dexamethasone dose and early postoperative recovery after mastectomy: a double-blind. Randomized Trial Anesthesiol.

[24] Nielsen S, Degenhardt L, Hoban B, Gisev N (2016). A synthesis of oral morphine equivalents (OME) for opioid utilisation studies. Pharmacoepidemiol Drug Saf.

[25] National Center for Injury Prevention and Control. CDC compilation of benzodiazepines, muscle relaxants, stimulants, zolpidem, and opioid analgesics with oral morphine milligram equivalent conversion factors, 2018 version. Atlanta, GA, USA.

[26] Voutilainen A, Pitkäaho T, Kvist T, Vehviläinen-Julkunen K (2016). How to ask about patient satisfaction? The visual analogue scale is less vulnerable to confounding factors and ceiling effect than a symmetric Likert scale. J Adv Nurs.

[27] Zoric L, Cuvillon P, Alonso S, Demattei C, Vialles N, Asencio G, Ripart J, Nouvellon E (2014). Single-shot intraoperative local anaesthetic infiltration does not reduce morphine consumption after total hip arthroplasty: a double-blinded placebo-controlled randomized study. Br J Anaesth.

[28] Kim MS, Koh IJ, Sohn S, Kang BM, Kwak DH, In Y (2019). Central sensitization is a risk factor for persistent postoperative pain and dissatisfaction in patients undergoing revision total knee arthroplasty. J Arthroplasty.

[29] Lei Y-T, Xie J-W, Huang Q, Huang W, Pei F-X (2020). The antifibrinolytic and anti-inflammatory effects of a high initial-dose tranexamic acid in total knee arthroplasty: a randomized controlled trial. Int Orthop.

[30] Matsuda M, Huh Y, Ji R-R (2019). Roles of inflammation, neurogenic inflammation, and neuroinflammation in pain. J Anesth.

[31] Tran J, Schwarzkopf R (2015). Local infiltration anesthesia with steroids in total knee arthroplasty: a systematic review of randomized control trials. J Orthop.

[32] Ikeuchi M, Kamimoto Y, Izumi M, Fukunaga K, Aso K, Sugimura N, Yokoyama M, Tani T (2014). Effects of dexamethasone on local infiltration analgesia in total knee arthroplasty: a randomized controlled trial. Knee Surg Sports Traumatol Arthrosc.

[33] Ng YCS, Lo NN, Yang KY, Chia SL, Chong HC, Yeo SJ (2011). Effects of periarticular steroid injection on knee function and the inflammatory response following unicondylar knee arthroplasty. Knee Surg Sports Traumatol Arthrosc.

[34] Agrawal Y, Smith RM, Garbuz DS, Masri BA (2018). Opioids in arthroplasty: mind the gap between North America and the rest of the world. J Bone Jt Surg Am.

[35] Nahhas CR, Hannon CP, Yang J, Gerlinger TL, Nam D, Della Valle CJ (2020). Education increases disposal of unused opioids after total joint arthroplasty: A cluster-randomized controlled trial. J Bone Jt Surg Am.

[36] Roshanaei-Moghaddam B, Pauly MC, Atkins DC, Baldwin SA, Stein MB, Roy-Byrne P (2011). Relative effects of CBT and pharmacotherapy in depression versus anxiety: Is medication somewhat better for depression, and CBT somewhat better for anxiety?. Depress Anxiety.

[37] Edwards RR, Haythornthwaite JA, Smith MT, Klick B, Katz JN (2009). Catastrophizing and depressive symptoms as prospective predictors of outcomes following total knee replacement. Pain Res Manag.

[38] Christensen CP, Jacobs CA, Jennings HR (2009). Effect of periarticular corticosteroid injections during total knee arthroplasty. A double-blind randomized trial. J Bone Jt Surg Am.

[39] Lunn TH, Kehlet H (2013). Perioperative glucocorticoids in hip and knee surgery–benefit vs harm? A review of randomized clinical trials. Acta Anaesthesiol Scand.

